# Treating Bacterial Infections with Bacteriophage-Based Enzybiotics: In Vitro, In Vivo and Clinical Application

**DOI:** 10.3390/antibiotics10121497

**Published:** 2021-12-06

**Authors:** Katarzyna M. Danis-Wlodarczyk, Daniel J. Wozniak, Stephen T. Abedon

**Affiliations:** 1 Department of Microbial Infection and Immunity, The Ohio State University, Columbus, OH 43210, USA; wozniak.1@osu.edu; 2 Department of Microbiology, The Ohio State University, Columbus, OH 43210, USA; abedon.1@osu.edu

**Keywords:** endolysin, EPS depolymerase, lysin, phage therapy, *Pseudomonas aeruginosa*, *Staphylococcus aureus*, tail spike protein, virion-associated peptidoglycan hydrolase

## Abstract

Over the past few decades, we have witnessed a surge around the world in the emergence of antibiotic-resistant bacteria. This global health threat arose mainly due to the overuse and misuse of antibiotics as well as a relative lack of new drug classes in development pipelines. Innovative antibacterial therapeutics and strategies are, therefore, in grave need. For the last twenty years, antimicrobial enzymes encoded by bacteriophages, viruses that can lyse and kill bacteria, have gained tremendous interest. There are two classes of these phage-derived enzymes, referred to also as enzybiotics: peptidoglycan hydrolases (lysins), which degrade the bacterial peptidoglycan layer, and polysaccharide depolymerases, which target extracellular or surface polysaccharides, i.e., bacterial capsules, slime layers, biofilm matrix, or lipopolysaccharides. Their features include distinctive modes of action, high efficiency, pathogen specificity, diversity in structure and activity, low possibility of bacterial resistance development, and no observed cross-resistance with currently used antibiotics. Additionally, and unlike antibiotics, enzybiotics can target metabolically inactive persister cells. These phage-derived enzymes have been tested in various animal models to combat both Gram-positive and Gram-negative bacteria, and in recent years peptidoglycan hydrolases have entered clinical trials. Here, we review the testing and clinical use of these enzymes.

## 1. Introduction

During WWII, penicillin was widely distributed to soldiers [1,2,3], resulting in the observation of the first “naturally” resistant *Staphylococcus* strains only 16 years (1944) after its discovery as the first antibiotic [4]. By 1948, up to 59% of the *Staphylococcus* isolates in hospital settings were penicillin-resistant [5,6]. Even though many new classes of antibiotics have been introduced since that time, their over prescription and misuse has resulted in today’s global antibiotic-resistance crisis [7,8]. Antibiotics are still very much part of standard-of-care treatments, however, and save many people from life-threatening bacterial infections. The year 1987 nevertheless marks the introduction of the last new class of antibiotics, lipopeptides [9,10], and for over 30 years no new antibiotics classes have been introduced to the market [7]. As we continue to experience a surge in bacterial antibiotic resistance, including pan- and multi-drug-resistant strains, there is a need for new antibacterials, especially ones with novel mechanisms of action.

**Figure 1:**
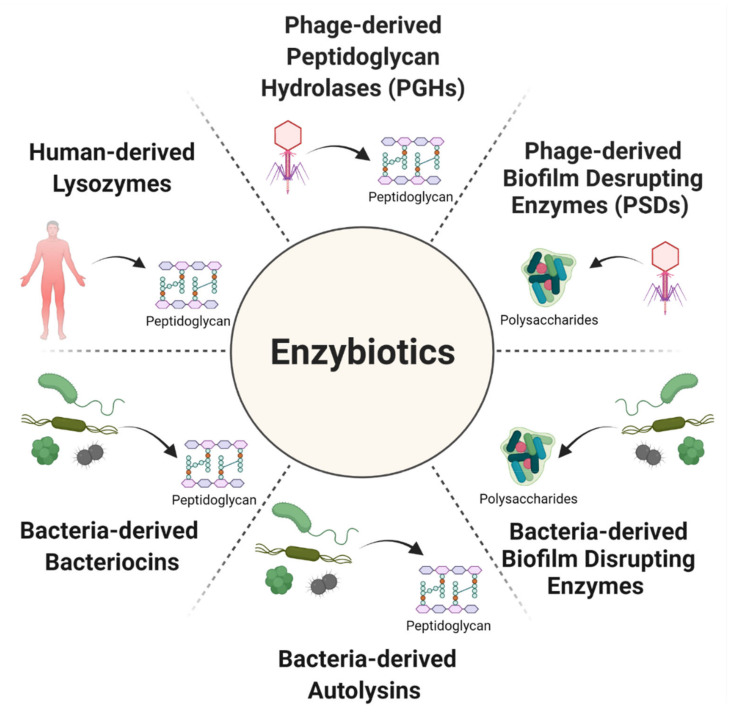
There are six main categories of enzybiotics. These include phage-derived enzymes: (1) peptidoglycan hydrolases (PGHs, which include endolysins and virion-associated peptidoglycan hydrolases) and (2) biofilm-disrupting enzymes (polysaccharide depolymerases, PSDs). In addition, among enzybiotics there are bacteria-derived enzymes: (3) biofilm-disrupting enzymes [19,20], (4) autolysins [21,22,23,24], which are bacterial enzymes that break down peptidoglycan to enable the separation of daughter cells following cell division, and (5) bacteriocins [24,25,26,27,28], which are bacteria-produced antibacterial proteins or peptides that inhibit the growth of closely related bacteria. Moreover, among enzybiotics are animal-derived enzymes including (6) the lysozymes found in natural body fluids, e.g., tears, saliva, milk, and mucous [24,27,29]. In addition, although not shown, there are enzybiotics with antifungal activities, i.e., fungal endoglucanases [30]. Arrows connect enzybiotic sources with targeted substrates. Figure created with BioRender.com (2020).

Enzybiotics are just such a promising alternative to standard antibiotics, possessing novel mechanisms of anti-pathogen action. The term ‘enzybiotics’ is a portmanteau word combining ‘enzyme’ and ‘antibiotic’. It was first used by Nelson et al. [11] in 2001 to refer to endolysins, often described simply as ‘lysins’, which are peptidoglycan hydrolases (PGHs) produced by bacteriophages or phages, bacterial viruses that can lyse and kill bacteria [12,13]. Especially in recombinant, purified forms, these lysins can be used as extracellularly acting antibacterial agents [14,15,16,17]. More recently, the term ‘enzybiotics’ has been used to describe not just lysins but also polysaccharide depolymerases (PSDs) [18], which are phage-derived enzymes that can also be used extracellularly to degrade bacterial polysaccharides, such as biofilm matrix, capsules, slime layers, or lipopolysaccharide (LPS). See Figure 1 for a summary of additional usage of this enzybiotic concept.

Here, we review the phage-encoded enzybiotics, PGHs and PSDs, which, to date, have been developed only from phages belonging to the *Caudovirales* order. We provide overviews of their in vitro properties, results of animal studies, and, for PGHs, clinical trials and human cases studies.

## 2. Phage Enzymes as the Basis of New Antibacterial Therapies

**Figure 2:**
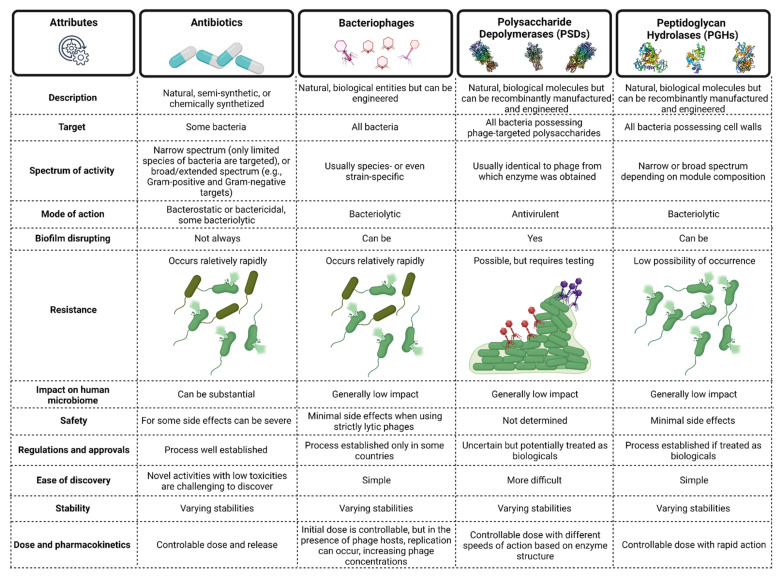
Typical attributes of antibiotics, bacteriophages, and two classes of phage enzymes, polysaccharide depolymerases (PSDs) and peptidoglycan hydrolases (PGHs). Figure created with BioRender.com (2020).

For over 100 years, whole phages have been successfully applied to treat various bacterial infections [31,32,33,34,35,36,37,38,39,40,41,42,43,44,45,46,47]. This includes infections caused by pathogens belonging to infamous ESKAPE group (*Enterococcus faecium*, Staphylococcus aureus, Klebsiella pneumoniae, Acinetobacter baumannii, Pseudomonas aeruginosa, and *Enterobacter* spp.) [48], whose members literally have escaped from currently available antibacterial therapies [49]. Many countries nevertheless are still cautious in their use of phages for human therapy due to challenges in pharmaceutical production, uncontrollable dosing, immunogenicity, possibility of horizontal gene transfer (particularly if temperate phages are used for therapy), resistance occurrence, legislative hurdles, and intellectual property issues [50,51,52,53]. Therefore, approximately 20 years ago, researchers started to explore phage-encoded antibacterial enzymes that can overcome some of these phage-therapy limitations. A comparison of the typical attributes of antibiotics, phages, PSDs, and PGHs [18,24,27,30,53,54,55,56,57,58,59,60,61,62,63,64,65,66,67,68,69] is found in Figure 2. Importantly, manufacturing PSDs and PGHs should not pose many hurdles since large scale recombinant protein production and purification strategies are well established.

### Roles of Polysaccharide Depolymerases and Lysins during Phage Infection Cycles

**Figure 3:**
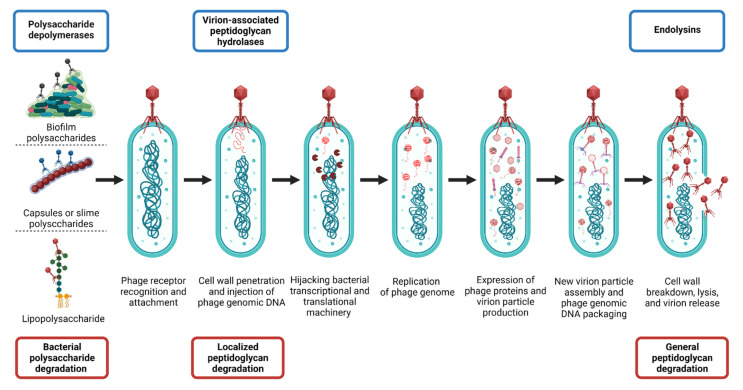
Lytic infection cycle of *Caudovirales* phages and involvement of phage-encoded enzymes, PSDs and PGHs, which in purified forms can possess extracellular antibacterial properties. Phage-encoded enzybiotics are listed in blue boxes on top and their activity is described in red boxes at the bottom. Figure created with BioRender.com (2020).

PGHs and PSDs play crucial roles during tailed-phage infections (Figure 3). First, PSDs [18,56,57,58,70,71,72] allow phages to degrade the bacterial barriers consisting of capsular polysaccharides (CPS), exopolysaccharides (EPS), and lipopolysaccharides (LPSs) [18], permitting virions to reach the compatible adsorption receptors found on cell-envelope surfaces [73,74,75]. Subsequently, the phage tail apparatus mechanically penetrates the bacterial cell wall, with the assistance of virion-associated peptidoglycan hydrolases (VAPGHs) that belong to the PGH group [15,64,76]. VAPGHs degrade the peptidoglycan layer only locally, generally without bacterial cell lysis [64], as at this step the phage needs to maintain the stability of the bacterial cell to continue its infection process [64]. In a pure, recombinantly manufactured form, VAPGHs, however, can lyse and kill bacteria upon direct contact with the cell walls [61], causing a variation in what is described in the phage literature as a lysis from without [76].

Next, phage genomic material is translocated into the bacterial cytoplasm, with bacterial transcriptional and translational machinery then hijacked by phage gene products in order to replicate phage genomes and assemble new virion particles. For dsDNA phages, holin proteins then perforate the bacterial inner membrane [77,78], allowing endolysins, the other type of PGHs, to gain access to the peptidoglycan layer and break down its structure. In the case of Gram-negative bacteria, phages also produce spanins that function to breach the bacterial outer membrane [79]. The result is a global degradation of the bacterial cell wall [76,80,81,82,83] and the release of new virion particles into the extracellular milieu. PSDs may also be used at this step to prevent the entrapment of newly released virions within biofilms [84].

## 3. Phage-Encoded Polysaccharide Depolymerases (PSDs)

As follows, we consider PSD enzymatic activities, discovery, in vitro properties, and in vivo testing.

### 3.1. PSD Substrate Diversity

**Figure 4:**
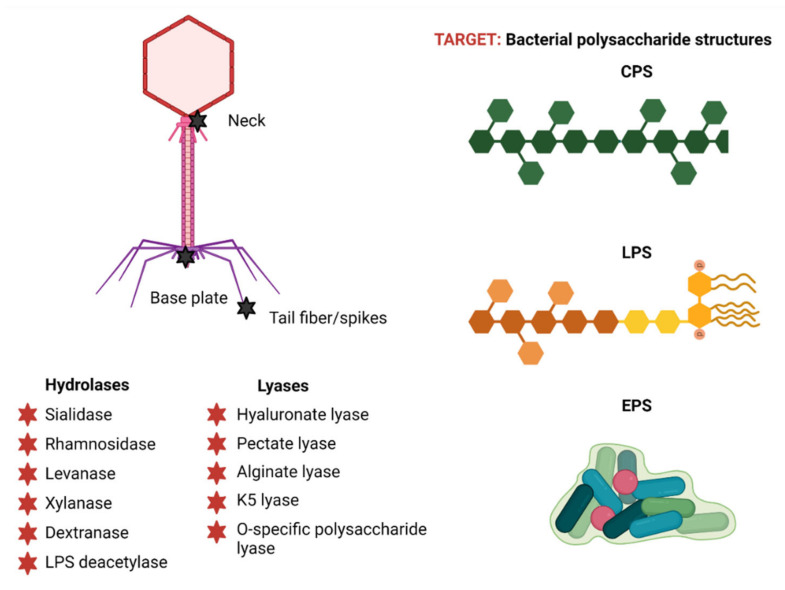
Phage-encoded polysaccharide depolymerases. PSDs virion-association is illustrated with black stars (upper, left), different enzyme classes are marked with red stars (lower, left), and bacterial polysaccharide targets are shown on the right. Abbreviations: (CPS) capsular polysaccharides, (LPS) lipopolysaccharides, (EPS) exopolysaccharides. Figure created with BioRender.com (2020).

PSDs collectively have a broad diversity in terms of their substrate specificities [18], although they can be generally differentiated into two classes: hydrolases and lyases [85] (Figure 4). The hydrolases catalyze the hydrolysis of glycosidic bonds [58] and currently include six groups: sialidases (hydrolyzing α-2,8-linkages in capsular polysialic acid) [86], rhamnosidases (cleaving α-1,3 O-glycosidic bonds between L-rhamnose and D-galactose) [87], levanases (hydrolyzing β-2,6-bonds between fructose monomers in levan) [88,89], xylanases (cutting β-1,4 bonds within xylan) [90,91,92,93], dextranases (cleaving α-1,6-linkages between glucose units in dextran) [94,95], and LPS deacetylases (deacetylating the O-antigen) [55,66,96]. The lyases instead cleave (1,4) glycosidic bonds by a β-elimination mechanism [97,98] and currently comprise five groups: hyaluronate lyase (cleaving β-1,4 bonds in hyaluronic acid) [99], pectate lyase (cleaving α-1,4 bonds of polygalacturonic acid) [100,101], alginate lyase (cutting α-1,4 bonds of alginate) [102,103], K5 lyase (cleaving α-1,4 bonds of *E. coli* K5 capsules) [104], and O-specific polysaccharide lyase (cleaving of O-specific antigen of LPS) [105].

### 3.2. Plaque Halo Zones

PSDs are often virion-associated as parts of tail fibers, tail spikes, baseplates, or neck proteins (Figure 4). They can also be released as free enzymes upon phage-induced lysis from within [18,58,106]. The latter occurs due either to the production by phage infections of excess proteins that do not end up being incorporated into virions, or the use of alternative start codons in their translation that result in the production of both soluble and virion-associated forms [18,58]. Both of these unincorporated forms can freely diffuse, causing polysaccharide degradation that is physically separated from PSD-producing phage infections [100,107], resulting in phage plaque-surrounding halo zones [75,100,105,106,108]. These halos may increase in size over a prolonged incubation time as free PSDs diffuse further within agar into bacterial lawns, although in some cases they can be absent, e.g., in the case of the *Salmonella* phage PVP-SE1 [58,109]. It is via the presence of these halo zones around phage plaques that PSDs are typically identified phenotypically.

### 3.3. Roadblocks to PSD-Gene Identification

Because of their diversity, an important bottleneck in PSD research and development is identification of their genes within phage genomes. In general, for many phage genes, putative functions cannot be assigned due to the limited homology to genes with known functions that are available in current databases. These genes, often described as ORFans [110,111,112], represent approximately 30% of the sequenced phage genomes [113], something that has been described as viral ‘dark matter’. In order to assign functions to these genes, there is often a need for experimental validation, e.g., [112], a process that can be laborious. The result is a low diversity of PSD genes that are available in databases and, therefore, limitations of new PSD gene discovery. Several groups, however, have attempted to bioinformatically identify PSD genes and their active domains [18,58] or construct protein prediction software, prediction models, or datasets, e.g., [114,115]. Nevertheless, it seems that the diversity of PSDs in nature may be much broader than what is currently known and available in databases.

### 3.4. Additional PSD Properties

Phages and their enzymes are part of the natural human microbiome [116,117,118] and serve as natural modulators of bacterial communities. They are generally considered to be safe and non-toxic [55,119]. PSDs also present high substrate specificity, leaving commensal flora unharmed. Bacterial resistance may evolve, however, due to modifications of bacterial polysaccharides, especially LPS or capsules. These modifications often lead to LPS or capsule defects that can result in lower bacterial fitness or virulence, e.g., [120,121,122,123].

In a handful of studies, anti-polysaccharide enzymes have also been used in purified [27,84,91,92,103,105,124,125,126,127,128,129,130,131,132,133,134,135] or phage-associated forms [127,136,137] towards the enhancement of antibacterial or anti-biofilm activities in vitro [122,136,138]. Phages can also be engineered with additional or new polysaccharide depolymerase genes that can be utilized during treatment, e.g., of bacterial biofilms [137]. Furthermore, phage depolymerases may act as antibiotic adjuvants, diagnostic tools, e.g., for targeting or detecting bacterial serotypes [66,84,105] or toward the production of oligosaccharides derived from polysaccharides [139,140].

Formulation possibilities can range from liquids to dry powders, ether of which can be stored for long periods [141,142,143]. Phage enzymes also tend to remain stable at wide pH ranges [55,105,144] as well as at both 4 °C and −80 °C.

### 3.5. In Vivo Charateristics

As antimicrobials, PSDs can be applied exogenously to degrade bacterial polysaccharides or potentially resensitize bacteria to antibiotics or the immune system [58,66], including to serum killing as well as phagocytosis. To our knowledge, however, the in vivo analyses of phage depolymerase efficacy have been limited to animal testing (Table 1). The published studies, however, present high animal survival rates when pretreated or treated with phage-derived recombinant and purified polysaccharide depolymerases, with no toxicity observed. PSDs in addition have been found to reduce bacterial virulence and levels of proinflammatory cytokines [145]. Like many other non-endogenous therapeutic proteins, i.e., biologics, PSDs might still induce allergic responses, although these have not been reported [146,147,148]. Phage-derived PSDs, therefore, represent a promising category of innovative antimicrobials to combat biofilms, reduce bacterial virulence, and improve immune-mediated killing [18,58,71,72,84,100,105,149].

## 4. Phage-Encoded Peptidoglycan Hydrolases (PGHs)

PGHs can be divided into two groups, ectolysins and endolysins [157]. Ectolysins (‘ecto’ referring to ‘outside’), also known as VAPGHs [15,64], are used by phages at the beginning of infection cycles to locally degrade bacterial peptidoglycan and allow the virus to inject its genome (Figure 3). Endolysins, in contrast, are responsible for the cell wall destruction that takes place during the phage-mediated lysis from within—‘endo’ meaning ‘within’—that occurs at the end of the phage lytic infection cycle (Figure 3).

### 4.1. Basic Characteristics

PGHs have become extensively studied for 20 years, e.g., [11,15,17,53,54,59,61,63,64,65,67,69,82,106,141,142,143,144,148,157,158,159,160,161,162,163,164,165,166,167,168,169,170,171,172,173,174,175,176,177,178,179,180,181,182,183,184,185,186,187,188,189,190,191,192,193,194,195,196,197,198,199,200,201,202,203,204,205,206,207,208,209,210,211,212,213,214,215,216,217,218,219,220,221,222,223,224,225,226,227,228,229,230]. They can be easily identified and prepared as pure recombinant proteins and have proven to be efficient in killing bacteria, including multi-drug-resistant variants [231,232,233,234]. They are rapid-acting [166,202,235,236,237,238,239] and stable over a range of pHs and temperatures [182,240,241]. PGHs also selectively target specific species of bacteria without harming commensal microflora [61,242,243]. Examples of pathogens against which they have been developed include *Bacillus anthracis* [180], *Clostridium* spp. [178,244], *Enterococcus faecium* [183], *P. aeruginosa* [205], *S. aureus* [186], *S. epidermidis* [188,245], *S. pneumoniae* [185], *K. pneumoniae* [184], and many others [27]. They can be used to treat biofilms [164,188,205,246,247,248,249,250], can be applied to mucosal surfaces [251], and are active against persister cells [188,252,253]. Furthermore, PGHs can serve as co-treatments with other antibacterial therapies, e.g., antibiotics or antibacterial enzymes of different origins [82,146,168,192,198,254,255,256,257,258,259,260,261,262].

In the course of in vivo studies (Section 5), PGHs have been found to display low toxicities [148,157,212,225,263,264,265,266,267,268,269,270,271,272]. Nevertheless, due to their proteinaceous nature, they may induce allergic reactions. PGHs can be applied not only in medicine [273] but also in the food industry [172,174,202,213,242,274,275,276,277,278,279,280], during biofuel production [210,281], in agriculture [282,283,284], and in veterinary medicine [258,285,286,287,288,289,290,291,292,293]. As with PSDs, they are suitable for a range of formulations and can be delivered via different routes, such as topical (creams, ointments, and gels), injections (intravenous or intraperitoneal), orally, transnasally, or vaginally [55,157,158].

### 4.2. Spectra of Activities and Resistance Evolution

The probability of bacterial resistance occurrence to PGHs is generally assumed to be low [142,157,190,198,212,222,252,253,273,294,295]. Some documentation exists of resistance to engineered forms or other types of PGHs, however, such as to human lysozyme [296,297,298,299] and *Staphylococcus simulans* secreted lysostaphin [300,301]. Resistance may occur due to, e.g., (1) modifications of peptidoglycan- and/or cell-wall-linked components, such as D-alanylation of teichoic acids, O-acetylation and *N*-deacetylation of peptidoglycan [298,299,302]; (2) mutations allowing the formation of monoglycine cross-bridges, which eliminate the target site for the bacteriocins’ catalytic site [273]; or (3) protection by small immunity proteins that bind to and inactivate the enzymes (pesticin secreted by *Yersinia pestis* cells [303]). The extracellular mode of action of PGHs, however, hampers the majority of the known intracellular antimicrobial resistance mechanisms, such as reduced membrane permeability, efflux pumps, and inactivation by cytoplasmic enzymes.

In comparison to whole phages (Figure 2), PGHs usually have a broader specificity [69], although not so broad as to disrupt normal microflora. This is because they target only a single bacterial macromolecule (peptidoglycan), which has a composition that is usually somewhat conserved at the species level versus the reliance by whole phages on multiple bacterial macromolecules for successful infection [69]. On the other hand, and unlike PSDs, unmodified PGHs usually have difficulty reaching their substrate targets in Gram-negative bacteria, as the cell walls there are surrounded by outer membranes [304], which serve as efficient barriers to protein diffusion [191,194,253,305]. For this reason, different approaches have been undertaken in recent years to support lysin activity against Gram-negative pathogens, including the use of chemical or physical methods to destabilize their outer membranes (e.g., high hydrostatic pressure, EDTA [217,306], citric acid, malic acid [216], carvacrol [307], cationic dendronized silver nanoparticles [308]) or synthetic biology and protein engineering to fuse outer membrane permeabilizing peptides with lysins (e.g., Artilysins^®^) [304]. Additionally, this approach allows to broaden the PGHs’ activity range, modify their specificity, as well as alter their other properties [53,61,62,67,191,194,203,215,252,253,303,305,309,310,311].

In recent years, also several lysin were discovered that have a natural capacity to penetrate the Gram-negative outer membrane. This property is called an intrinsic antibacterial activity [304]. Some of these enzymes possess unusually broad activity spectra, spanning from Gram-negative to Gram-positive pathogens [304,312,313,314,315]. Their mechanism of action is not fully understood, but it is hypothesized that it is correlated with a self-promoted uptake mechanism by a C-terminal amphipathic helix that interacts with the outer membrane, while the *N*-terminal enzymatic domain degrades peptidoglycan [304]. It is possible that this C-terminal amphipathic helix acts similarly to phage spanin in spanin-less phages, disrupting the bacterial outer membrane from within [79,304]. The uptake can be further enhanced by a positively charged hexaHis-tag [312,313,316].

### 4.3. Structure and Mode of Action of Phage-Derived Peptidoglycan Hydrolases

Although they have a conserved biological function, i.e., the degradation of bacterial cell wall material, PGHs nonetheless are diverse in their structures, catalytic activity, specificity, and enzyme kinetics [62,317]. This is due in part to differences between the Gram-negative and Gram-positive bacterial cell wall structure. Enzymes targeting Gram-negative bacteria usually have a globular structure consisting of an enzymatically active domain (EAD) [203], while enzymes targeting Gram-positive bacteria have a modular structure composed of an *N*-terminal EAD, flexible interdomain linker region, and C-terminal cell-wall-binding domain (CBD) [54,158,170,171,318,319]. Exceptions, however, exist, such as the presence of two EADs. Additionally, mycobacteriophages produce two forms of EAD lysins, lysin A (peptidoglycan hydrolase) and lysin B (mycolyl arbinoglacan esterase) [69,320,321].

**Figure 5:**
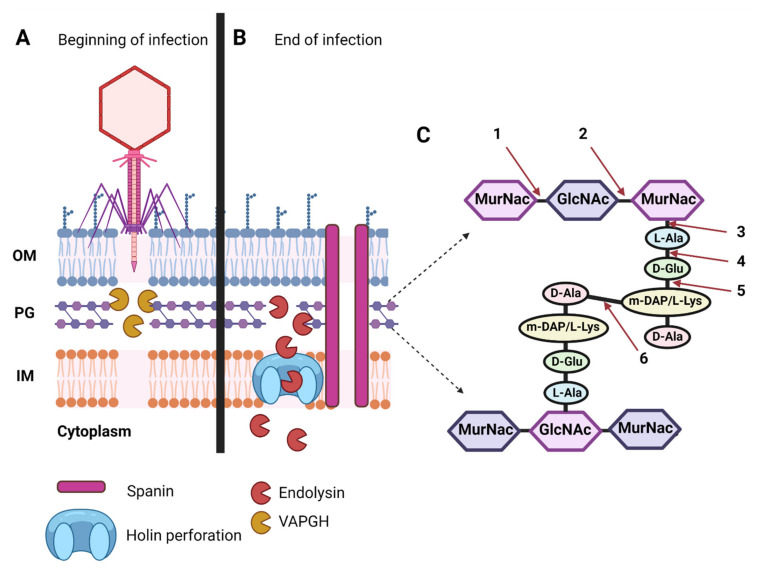
Mode of action of the peptidoglycan-degrading enzymes. (**A**) Schematic representation of Gram-negative bacteria cell wall structure. OM represents bacterial outer membrane, which is absent in the case of Gram-positive bacteria. PG is the peptidoglycan layer and IM stands for inner membrane. At the beginning of phage infection cycles, phage virions mechanically perforate bacterial cell walls with their tail structures. This process may be assisted by virion-associated peptidoglycan hydrolases that digest through bacterial PG. (**B**) At the end of phage infection cycles, with holin-dependent translocation mechanisms, holins are distributed in the IM, creating 2D aggregates called “rafts”, which lead to a collapse in the proton motive force and lesion (blue doughnut) formation (perforation of the inner membrane). Endolysins (red packman symbols) can then pass to access the peptidoglycan layer. Additionally, another set of proteins called spanins (pink bars spanning from IM to OM) is required to disrupt the outer membrane in the lysis of Gram-negative bacteria. Finally, bacterial cell wall lysis occurs. (**C**) Bacterial peptidoglycan structure magnification. The PG layer is built with alternating *N*-acetylmuramic acids (MurNAc) and *N*-acetylglucosamines (GlcNAc), which are crosslinked by peptide stems. The interpeptide bridge consists of a diamino acid (m-DAP) that is directly cross-linked to the terminal D-Alanine (D-Ala) of the opposite peptide chain. Red arrows represent possible PGH (endolysin or VAPGH) cleavage sites, including: (1) *N*-acetyl-β-D-muramidase, (2) *N*-acetyl-β-d- glucosaminidase, (3) *N*-acetylmuramoyl-L-alanine amidase, (4) l-alanoyl-d-glutamate endopeptidase, (5) interpeptide bridge-specific endopeptidase, and (6) γ-d-glutaminyl-l-lysine endopeptidase. Figure created with BioRender.com (2020).

CBDs are responsible for the noncovalent binding to different epitopes on bacterial cell wall surfaces, such as teichoic acids, peptides, or carbohydrates, facilitating the enzymatic activity of EADs [54,171,177,322,323]. Like PSDs, the EADs of PGHs also have different catalytic activities, targeting different bonds in the peptidoglycan structure (Figure 5C). They are divided into three distinctive classes: glycosidases, amidases, and endopeptidases. Glycosidases cleave β-1,4 glycosidic bonds linking *N*-acetylmuramic acids (MurNAc) and *N*-acetylglucosamines (GlcNAc) in the peptidoglycan structure and include (1) *N*-acetyl-β-d-muramidases (similar to lysozyme) that cleave the *N*-acetylmuramoyl-β-1,4-*N*-acetylglucosamine bond, one of the two alternating glycosidic bonds of the glycan strand, and (2) *N*-acetyl-β-d-glucosaminidases that hydrolyze the other glycosidic bond (*N*-acetylglucosaminyl-β-1,4-*N*-acetylmuramine), which is found between the peptidoglycan sugars. Amidases, i.e., *N*-acetylmuramoyl-L-alanine amidases, cleave amide bonds between sugar (glycan, MurNac) and L-alanine, the first amino acid in the side peptide stem. Endopeptidases cleave between amino acids in the side stem peptide. These include l-alanoyl-d-glutamate endopeptidases (VANY), c-d-glutamyl-m-diaminopimelic (DAP) acid peptidase, d-Ala-m-DAP endopeptidase, and d-alanyl-glycyl endopeptidase (CHAP), etc. [170,171,207,323,324]. 

There are different models for the export of phage endolysins during infection cycles. These generally are divided into two groups: holin-dependent and holin-independent, the latter as found in association with what are described as pin-hole holins. For further discussion, see, e.g., [14]. In Figure 5, we represent only the holin-dependent mechanism.

## 5. Clinical Trials and Case Studies

Phage PGHs are eligible for FDA (U.S. Food and Drug Administration) fast-track status. This allows the expedition of their review toward approval as drugs for treating serious or life-threatening conditions and otherwise to fulfill unmet medical needs. Currently, seven clinical trials have been launched, all of which target *S. aureus*. In this section, we describe in vitro, clinical, and also pre-clinical (animal testing) analyses of these lysins: P128 ectolysin (VAPGH) (Section 5.1) and endolysins *N*-Raphasin^®^ SAL200 (Section 5.2), CF-301 (Section 5.3), and Staphefekt SA.100 (Section 5.4). The details of the clinical trials are summarized as well in Table 2.

### 5.1. P128: Anti-Staphylococcal Engineered VAPGH

P128 is an engineered chimeric protein. It was created by the fusion of Lys16, an *N*-terminal truncated form of a phage tail-associated ectolysin (VAPGH, gp56) that is encoded by the strictly lytic *Staphylococcus* phage K, and SH3b, a lysostaphin cell-wall-binding domain encoded by *S. simulans* [326]. Lys16 consists of a CHAP domain (Cysteine, Histidine-dependent Amidohydrolase/Peptidase) with muralytic activity [326] that can cleave the pentaglycine cross-bridge of peptidoglycan [295,327,328]. These pentaglycines are absent in genera other than staphylococci. P128, therefore, is highly specific to the *Staphylococcus* genus [329]. P128 otherwise is a potent bactericidal treatment able to target antibiotic-resistant clinical *S. aureus* strains as well as coagulase-negative (CoNs) staphylococci, such as *S. epidermidis*, *S. haemolyticus*, and *S. lugdunensis* [330]. It is a fast-acting enzyme that can target metabolically active as well as metabolically inactive cells as found either in a planktonic or biofilm-associated state [329].

#### 5.1.1. P128 In Vitro Activity Analysis

Various in vitro analyses have shown that P128 can be highly effective against diverse staphylococcal strains. In Paul et al. [326], P128 was shown to be able to kill >99.9% of the *S. aureus* cells at a concentration of ≥2.5 μg/mL. When a panel of 3000 *S. aureus* isolates were tested, P128 presented a very broad range of activity, killing 99.9% of these strains, including MRSA (methicillin-resistant *S. aureus*), MSSA (methicillin-sensitive *S.* *aureus*), and mupirocin-resistant strains (at 10 μg/mL dose) [326] (see also [331]). Additionally, Poonacha et al. [330] reported that 90% of 62 clinical CoNs were P128-sensitive at P128 concentrations of 16 and 32 μg/mL. The broad activity of this drug is probably due to its ability to cleave serine- or alanine-containing pentapeptides [295,330]. Furthermore, MRSA and MSSA 48-h-old biofilms could be reduced up to 95.5% with ≥12.5 μg/mL doses [332] (see also [329]), as well as 72-h-old CoNs-related biofilms formed on the surface of 96-well plates or catheters [328,330]. P128 has also been shown to be effective in vitro in combination with standard-of-care anti-staphylococcal antibiotics [329,330]—such as daptomycin [328,329,330], vancomycin [329,330], linezolid [329,330], gentamicin [329], ciprofloxacin [329], oxacillin [328], and cephazolin [328]—for treatment of sensitive and resistant staphylococcal isolates.

#### 5.1.2. P128 In Vitro and Ex Vivo Stability and Lack of Cytotoxicity

P28 has been found to be highly stable in the presence of the divalent cations, calcium, magnesium, and zinc, as well as EDTA, human serum, plasma, whole blood, hyper-immune sera [333], and at a fairly substantial range of temperatures (37–70 °C) [327]. In addition, no cytotoxicity was observed against HEp2 and Vero cell lines [333].

#### 5.1.3. P128 Resistance

Sundarrajan et al. [295] identified bacterial resistance occurrence in response to P128 treatment and to the Lys16 domain alone, probably due to alterations in the peptidoglycan cross-bridges (only a single glycine versus pentaglycine cross-bridges in wild type). Bacterial mutants, resistant to Lys16, however, displayed reduced fitness, as indicated by slow growth rates in vitro. These alterations in bacterial fitness may lead to reduced pathogenicity, reduced pathogen survival, or reduced pathogen spreading within communities [295]. Often, mutations were also unstable, with mutants reverting to the wild type. P128-resistant mutants of *S. aureus* and *S. epidermidis* also become sensitive to the β-lactam antibiotics, vancomycin, linezolid, and daptomycin [328], which might occur due to changes in the bacterial cell wall structure [328].

The frequency of *S. aureus* mutation to resistance to P128 is comparable to the lower range value (1 × 10^−7^) of lysostaphin [295,334].

#### 5.1.4. P128 Animal Testing

Several pre-clinical in vivo P128 efficacy studies have been done using mice, rats, and dogs.

Paul et al. [326] tested this enzyme using a rat nasal colonization model. Healthy female Wistar rats (6–7 weeks old) were intranasally inoculated with 10 µL of 2 × 10^8^ to 5 × 10^8^ cells/μL of the MRSA USA300 strain. P128 was administrated twice daily, 24 h post-infection, in the form of a hydrogel (50 mg/dose containing 100 μg P128). Three days of treatment completely decolonized the nasal tissue in 44.4% of the rats, and, for the rest of the animals, a 2-log reduction in the bacterial burden was observed relative to the negative-treatment control.

Junjappa et al. [286] tested P128 hydrogels on 17 dogs with staphylococcal pyoderma. The treatment was applied daily for 8 days, resulting in complete recovery and no recurrence of symptoms for 2 months.

Sriram et al. [335] explored a bacteremia neutropenic mouse model to evaluate P128 as well as its pharmacokinetics. The half-life of P128 was determined as 5.2 h (30 mg/kg dose) to 5.6 h (60 mg/kg dose), and the maximum bactericidal effect was observed after 30 min of treatment for all the tested doses (10, 30, and 60 mg/kg).

Nair et al. [328] tested the efficacy of P128 in combination with oxacillin against MRSA bacteremia in a mouse model. Female BALB/c mice were infected with 10^9^ CFU of USA300 strain via IP injection. Next, 2 h post-infection, a sub-minimal dose of P128 (2.5 mg kg^−1^, IP) and/or oxacillin (four doses in 4 h intervals of 100 mg kg^−1^, intramuscularly) was administrated. The untreated control group died within 12 h (>80%), while antibiotic and P128 administrated alone could protect 31% and 50% of mice, respectively. Co-treatment, however, protected 81% of the animals up to 72 h, indicating a therapeutic potential of dual treatment.

Channabasappa et al. [336], in a MRSA bacteremia mouse model, saw similar results. BALB/c mice were challenged with 10^9^ CFU of the USA300 strain, IP. Next, the animals were treated with a single dose of P128 (0.2 mg/kg, IP) and/or with sub-inhibitory doses of two antibiotics, vancomycin (two doses of 55 mg/kg, subcutaneously, 12 h intervals) or daptomycin (two doses of 12.5 mg/kg, also subcutaneously with 12 h intervals). The absence of treatment resulted in 88% dead animals. Monotherapy resulted in 31%, 46%, and 46% of survival, respectively, for P128, vancomycin, and daptomycin. Co-treatments of P128 and vancomycin or P128 and daptomycin increased mice survival to 85% and 88%, respectively.

Channabasappa et al. [337] employed a MRSA bacteremia rat model to further test P128 antibacterial efficacy. The animals were inoculated with 10^9^ CFU of the USA300 strain intravenously with P128 delivered 2 h post-infection by intravenous bolus administration via the tail vein or by 1 h infusion by the jugular vein. The untreated group had 80–100% mortality by day 14, while the survival in the treatment group was dose-dependent. The bolus treatment with 0.5 mg/kg or 2.5 mg/kg resulted in 54% and 100% survival, respectively, by day 14. The intravenous infusion, 2 h post-infection, with 10, 2.5, or 0.5 mg/kg of P128 resulted in 84%, 66%, or no effect, respectively. In contrast, 84% of the rats died in the control untreated group. The treatment with P128 also minimized renal abscess occurrence or abscess size.

#### 5.1.5. P128 Clinical Trial

To date, one phase I/II P128-based clinical trial has been conducted (Table 2) (ClinicalTrials.gov identifier NCT01746654). The safety, tolerability, and efficacy in healthy individuals, or various patients that are nasal carriers of *S. aureus* or MRSA strains, were evaluated, with the drug administrated intranasally. The final results of the trial are not available to the public at this moment. The initial results, however, suggested that P128 was well tolerated, with the nasal *S. aureus* burdens reduced.

### 5.2. N-Rephasin^®^ SAL200: Anti-Staphylococcal Recombinant Endolysin

N-Rephasin^®^ SAL200 (SAL200) is a recombinant SAL-1 endolysin that is naturally encoded by staphylococcal phage SAP-1 [164,338,339]. It is active against both planktonic and biofilm-embedded *S. aureus* strains, including MRSA [157]. The N-Rephasin^®^ SAL200 stabilizing and enhancing formulation for human application was created in 2013 by Jun et al. [164], consisting of SAL-1 purified by a two-step chromatography, 10 mM of calcium ions, 0.1 (*w*/*v*) Poloxamer 188, 0.01 M L-histidine at pH 6.0, and 5% (*w*/*v*) sorbitol. Antibacterial activity was stable for 8 weeks at 4 °C and up to 4 h with constant vigorous agitation.

#### 5.2.1. N-Rephasin^®^ SAL200 In Vitro Analysis

A SAL200 preparation was found to be highly effective against all 425 clinical *S. aureus* isolates tested, including 336 MRSA strains, as well as against pathogens in planktonic and biofilm-related life styles [164]. SAL200 was active not only in broth but also in serum [164].

Kim et al. [256] also assessed the in vitro efficiency of co-treatments with SAL200 and anti-staphylococcal antibiotics (nafcillin and vancomycin). A minimum inhibitory concentration was established between 0.8 and 1.6 µg/mL depending on the *S. aureus* strain being tested. When this combination treatment was applied against MRSA or MSSA strains, indifferent or synergistic effects were observed. At sub-MIC antibiotic dosages, the SAL200-antibiotic combination rapidly killed the bacteria. However, the bacterial culture subsequently regrew for all the *S. aureus* strains tested.

#### 5.2.2. N-Rephasin^®^ SAL200 Animal Testing

Jun et al. [164] used an MRSA ICR mouse model for testing SAL200 efficiency. The animals were infected with *S. aureus* SA2 isolate (1 × 10^8^ CFU) intravenously and subsequently treated with SAL200 at a 12.5 mg/kg or 25 mg/kg dose. These treatments consisted of intravenous SAL200 applications at 1 h, 25 h, and 49 h post-infection. The result was a significantly reduced bacterial burden in the bloodstream and splenic tissues (at least a 5-log reduction in comparison to the untreated controls) as well as increased mouse survival over a 5-day period: 9/15 mice died in the control group, 1/15 in the treatment group with a low dose, and no mortality was observed in the higher dose group. Additionally, 2 days post-infection, the negative-treatment control group exhibited several side effects, including erythema of the lid margin, decreased locomotor activity, loss of fur, ptosis, to piloerection and circling, while the mice in both treatment groups behaved normally for the entire experimental period.

The same group [271] also found later that, when SAL200 alone was administrated intravenously into the tail vein of dogs and rats as a single dose, no signs of toxicity in the central nervous system were observed. Only mild adverse effects on respiratory and cardiovascular system functions as well as animal behavior were observed in the case of the dogs. These mild adverse effects included subdued behavior, prone posture, irregular respiration, vomiting, and transient changes in cardiovascular function (one dog, injected with 25 mg/kg). The abnormal clinical signs were not observed at any other time during the recovery period, beginning ~10 days post-injection. In a subsequent part of this study, Jun et al. also considered the impact of repeated SAL200 application, particularly in terms of immune-system interactions. Anti-SAL-1 antibodies were found to be absent from the blood samples at day 14, although they appeared at day 28 in the rats. In the dogs, anti-SAL-1 antibodies instead were detected at day 14. Mild side effects were observed in the repeated-dosing dog model, correlated with decreased C3 complement levels in the blood. It is not clear, however, what stimulated this innate immune system response; i.e., it might have occurred due to the presence of endotoxins in the SAL200 formulation (<0.5 endotoxin unit/mg), or, rather, due to the presence of the enzyme itself. All the experiments were performed according to general laboratory practice and served as a basis for an exploratory phase I clinical trial [157], as presented below.

Kim et al. [256], in an in vivo *S. aureus* bacteremia mouse SAL200-treatment model, observed decreased bacteria counts in the blood by ~2.2 log and ~3.4 log for the MRSA and MSSA strains, respectively, both 1 h post-treatment. When antibiotics (vancomycin or nafcillin) were co-administrated 1 h post-infection, bacteria counts in the bloodstream were reduced by 2.1 and 1.6 log for the MRSA and MSSA strains, respectively, in comparison to the untreated controls. In the same study, with the use of a wax moth model, the SAL200 and antibiotic (cefazolin or vancomycin) co-treatment also improved the survival of *S. aureus* infected larvae by 33.3% for MRSA and 73.3% for MSSA infection in comparison to the untreated group (96 h post-infection). The treatment with just antibiotics increased the survival instead by 6.7% and 46.7%, respectively, for MRSA and MSSA infections in comparison to the untreated group.

#### 5.2.3. N-Rephasin^®^ SAL200 Clinical Trial

In 2013, a phase I randomized, double-blind, placebo-controlled clinical trial was started [157] (Table 2; ClinicalTrials.gov identifier NCT01855048) to evaluate the safety, pharmacokinetics, and pharmacodynamics of intravenous infusions of SAL-1 using single ascending doses (0.1 mg/kg, 0.3 mg/kg, 1 mg/kg, 3 mg/kg, or 10 mg/kg) administrated to healthy male individuals. This was the first human phase I study of a phage endolysin using intravenous administration. No severe adverse events were seen during observations over the 50 days following SAL200 application and the drug otherwise was tolerated [157]. Mild adverse effects were seen, however, e.g., fatigue, rigors, headache, and myalgia, but were both self-limited and transient. Moreover, no changes in patients’ vital signs, ECG, serum chemistry, hematology, or urinalysis test were observed.

The SAL200 half-life in serum ranged from 0.04 to 0.38 h, and maximum concentration in serum was reached between 0.25 to 1.0 h depending on the dose [157]. Pharmacodynamic properties were also evaluated, but this was done ex vivo, measuring antibacterial activity of blood spotted on an *S. aureus* lawn as compared with a standard series of SAL200 dilutions [157]. The resulting antibacterial blood activity was approximately proportional to the drug dose, and the minimal bactericidal concentration was established at 0.078 µg/mL. The observed clearance of SAL200 from patients was potentially due to proteolysis by plasma proteases. As expected, based on previous monkey studies [272], a humoral immune response to SAL200 was found to be present. Protein-induced antibody production was observed in serum (2 to 12 μg/mL) and was proportional to drug dose [157].

In 2017, a phase II randomized, double-blind, placebo-controlled clinical trial was launched (ClinicalTrials.gov identifier NCT03089697) but terminated prior to completion: “Enrollment into this study was terminated by the Sponsor prior to completion for strategic reasons (to initiate clinical development abroad)”. Several minor side effects occurred in 83.33% of SAL200-treated patients (10 out of 12 tested subjects), including e.g., anemia, chills, back pain, headache, gastrointestinal disorders. In the placebo group, 12 out of 13 patients (92.31%) experienced various minor side effects. Importantly, two patients out of 12 SAL200-treated (16.67%) developed severe side effects, i.e., pneumonia or respiratory failure. In the placebo group, also two patients out of thirteen (15.38%) developed serious side effects, i.e., cardiac disorder due to acute infection, or respiratory failure type 2. It is not clear why side effects occurred in this clinical study. No results are published except general information placed on ClinicalTrials.gov. Therefore, it is unclear if the side effects occurred due to reaction to SAL-1, level of endotoxins in SAL200 formulations, or maybe due to other co-existing patients morbidities, the infection itself, or other reseaons.

### 5.3. CF-301: Anti-Staphylococcal Recombinant Endolysin

CF-301, also called PlySs2 or exebacase, is a *Streptococcus suis* prophage-encoded endolysin [143,340]. CF-301 has a modular structure, comprised of an *N*-terminal cysteine-histidine aminopeptidase (CHAP) catalytic domain and a C-terminal SH3b cell wall binding domain [143]. This enzybiotic has a broad lytic activity against various Gram-positive pathogens including *S. aureus* [340], *S. suis*, *Listeria* spp., *Staphylococcus simulans*, *S. epidermidis*, *Streptococcus equi*, *Streptococcus agalactiae* (group B streptococcus), *S. pyogenes*, *Streptococcus sanguinis*, group G and E streptococci, and *Streptococcus pneumoniae* [143,340]. CF-301 also has been found to possess anti-biofilm, anti-persister cell, and anti-small-colony variants activities against *S. aureus* [341]. We report here on three clinical trials, phase I, II, and III, that have employed this endolysin, although, as above, we begin with consideration of additional in vitro as well as in vivo properties.

#### 5.3.1. CF-301 In Vitro and Ex Vivo Analysis

Gilmer et al. [143] found that MRSA and *S. pyogenes* strains could be reduced by 5 logs and 3 logs, respectively, within 1 h post-CF-301 application. They also found, as with other PGHs, that CF-301 is suitable for various formulation strategies and delivery routes. In addition, CF-301 was found to be stable at a relatively wide range of temperatures (50 °C for 30 min, 37 °C for >24 h, 4 °C for 15 days, and −80 °C for >7 months) and pHs (between pH 6 and 9.7, with optimum at pH 8), while also being relatively salt- and DTT- (dithiothreitol) resistant. Exposure of MRSA and *S. pyogenes* to increasing dosages of endolysin (1/32× to 4× MIC) over an 8-day period, moreover, resulted in no resistance occurrence. Schuch et al. [340] also found that CF-301 treatment could reduce *S. aureus* viable counts (62 strains tested) by ≥3 logs within 30 min in broth, a rapidity of action consistent with that of other PGHs. Furthermore, they confirmed that CF-301 has a low resistance profile.

CF-301 was also found to work in combination with other antimicrobial agents, such as lysostaphin [340,341], vancomycin, daptomycin, oxacillin, nafcillin, and cefazolin [342]. Bacterial cells could be re-sensitized to antibiotics in the course of CF-301 treatment by enhancing daptomycin and vancomycin binding to bacterial cell walls and membrane [340]. Oh et al. [343] similarly were able to enhance daptomycin activity against *S. aureus* when used in combination with sub-MIC concentrations of CF-301 (as low as 0.001× to 0.01× MIC, corresponding to ∼1 to 10 ng/mL). They concluded in their study that CF-301 caused an increase in *S. aureus* membrane permeability, dissipation of membrane potential, and inhibition of virulence phenotypes, including agglutination and biofilm formation.

Schuch et al. [341], in addition, found that mature biofilms of *S. pyogenes* and *S. agalactiae* could be disrupted with a MBEC_90_ (minimum biofilm-eradicating concentration) ranging from 0.25 to 8 μg/mL. *S. aureus* biofilms formed on different surfaces, including polystyrene, glass, surgical mesh, catheters, or in the presence of synovial fluid, were also successfully degraded or even completely removed with the use of this pure recombinant protein. The latter included *S. aureus* biofilms formed on catheters, which could be completely removed within 1 h, with all released bacterial cells killed within 6 h.

Indiani et al. [344] presented evidence of the enhanced potency of CF-301 in combination with human blood components (serum and albumin) and lysozyme, where the latter factors otherwise had no impact on staphylococcal strains (four clinical MSSA, seventy-five clinical MRSA isolates, and twenty-two additional vancomycin-resistant, linezolid-resistant, and daptomycin-resistant *S. aureus* strains). This shows a great potential for this lysin to be used as an adjunct therapeutic toward treating severe systemic infections in humans. Importantly, albumin also substantially increases CF-301 activity, which distinguishes this lysin from small molecule antibiotics.

#### 5.3.2. CF-301 Animal Testing

Gilmer et al. [143] infected FVB/NJ mice with MRSA, *S. pyogenes*, or a mix of both via IP injection (∼5 × 10^5^ CFUs/mL with 5% hog gastric mucin in saline). The animals become bacteremic within 1–3 h and infection spread through different organs. CF-301 treatment was applied 3 h post-infection, delivered as a single dose (2–4 mg/mL), and it successfully rescued 89% of the MRSA-infected mice and 94% of *S. pyogenes*-infected mice. In the negative-treatment control group, only 6% and 7% mice survived MRSA and *S. pyogenes* infection, respectfully. When mixed infections were introduced, only 4% of mice survived in control group versus 92% in CF-301 treated over the 10-days period.

Schuch et al. [340], in a MRSA-related BALB/c mouse bacteremia study, found that CF-301 exhibited a dose-dependent activity. Mice were infected with 0.5 mL of 7.5 × 10^6^ to 1 × 10^8^ CFU MRSA in 5% mucin, interperitoneally. CF-301 treatment was administrated intraperitoneally 3 h post-infection at concentrations ranging from 0.25 to 5 mg/kg. High levels of protection (70% of mice) were achieved with 5 mg/kg over 24 h. Additionally, endolysin treatment resulted in a half-log decrease in bacterial numbers in blood within 15 min and 2-log decrease with 60 min of treatment.

Asempa et al. [345] determined the combined impacts of CF-301 and subtherapeutic daptomycin treatments against *S. aureus* in a neutropenic murine thigh infection model. Daptomycin treatment alone applied 2 h post-infection resulted in a mean growth of only 0.39  ±  1.19 log in CFUs/thigh in comparison to 8.28  ±  0.47 log CFUs/thigh in the untreated control group. Treatment with CF-301 alone resulted in mean growth instead of only 0.76  ±  1.24 log CFUs/thigh for 15 mg/kg dose while a 90 mg/kg dose reduced bacterial growth to −0.26  ±  1.25-log of CFUs/thigh. Co-treatment with 15 mg/kg CF-301 and daptomycin resulted in a mean growth also of −1.03  ±  0.72 log CFUs/thigh. Introducing higher doses of CF-301 did not yield further killing in co-treatment. Thus, CF-301 at the higher dose, when used alone, appeared to be superior to daptomycin treatment, with no improvements seen with combined treatment, and with superior effectiveness seen when combining daptomycin treatments with the lower CF-301 dose.

In the MRSA septicemia model, conducted by the same group [345], a high MRSA inoculum (1 × 10^9^ CFU) was administrated IP, followed by treatment with CF-301, daptomycin, or co-treatment using both drugs, delivered 2 h post-infection. Treatment with CF-301 alone rescued 17–50% of mice at 72 h post-infection, while antibiotic treatment alone resulted in only 7–31% survival. Co-treatment, however, improved mice survival to 82–90%, clearly presenting synergistic effect of both drugs.

#### 5.3.3. CF-301 Clinical Trials

In 2015, CF-301 was subjected to a phase I clinical trial (Table 2; ClinicalTrials.gov identifier NCT02439359) focusing on the safety and tolerability of single intravenous dose as administrated to healthy individuals. CF-301 was found generally to be safe and well tolerated, with no serious side effects observed. Inflammatory responses to the drug were evaluated using a range of inflammatory markers, e.g., high-sensitivity C-reactive protein, the erythrocyte sedimentation rate, and complement factors Bb, C3a, C5a and CH50, but no differences were observed between placebo- and CF-301-treated groups [268]. Furthermore, no clinically relevant changes were found in heart rate, QT interval in electrocardiograms, or either systolic or diastolic blood pressure [269].

CF-301 was the first phage-derived PGH to be entered into a phase II clinical trial (ClinicalTrials.gov identifier NCT03163446), which was a randomized, double-blind, placebo-controlled, superiority-design, first-in-patient, proof of concept study conducted between 2017 and 2019 in 11 countries [325]. The trial involved the treatment of 121 patients with and without antibiotic co-treatment for bacteremia, including endocarditis, of which 116 patients had confirmed *S. aureus* infections, including by MRSA and MSSA strains [346]. The efficacy analysis of CF-301 treatment showed a greater frequency of clinical responders for MRSA infections on a day 14 with the co-treatment subgroup than with the comparison, antibiotic-only subgroup (92.6% vs. 75%). In MSSA subgroup, however, little difference was observed, i.e., 88.6% versus 90%, respectively, perhaps because the response to antibiotic treatment alone was already quite high with this subgroup [325]. Importantly, in MRSA subgroup, mortality rate at day 30 was 3.7% in co-treatment subgroup versus 25% in antibiotic-alone subgroup. The results of this trial are considered, however, more as proof-of concept rather than confirmatory.

Note, that CF-301 endolysin was considered to be generally safe and well tolerated by the patients, with no hypersensitivity reactions. In addition, although 20.8% and 14.9% of the patients had preexisting CF-301-antibodies in the co-treatment and antibiotics-alone subgroups, respectively, these antibodies did not affect the efficacy or safety outcomes of CF-301 treatments.

In 2019, a phase III clinical trial was launched (ClinicalTrials.gov identifier NCT04160468) evaluating the efficacy and safety of a single dose of CF-301 in addition to standard-of-care antibacterial therapy in adult individuals with bloodstream infections (bacteremia), again including endocarditis. The results are not yet publicly available. Anastasiou et al. [347] reported at p. S320, however, that there were “42.8% higher clinical responder rates with a single dose of exebacase used in addition to standard of care antibiotics (SOC) vs. SOC alone for the treatment of methicillin-resistant *S. aureus* (MRSA) bacteremia including endocarditis”.

### 5.4. Staphefekt SA.100: Anti-Staphylococcal Engineered Endolysin

Staphefekt SA.100 is an engineered phage endolysin with endopeptidase and putative amidase activity. It is used to treat skin infections caused by *S. aureus*, including by MRSA and MSSA strains [212]. It is registered as a medical device in Europe (class 1) and available in pharmacies there as an over-the-counter medicine in the form of a cream and gel [348]. Staphefekt is somewhat specific to *S. aureus* and does not affect commensal bacteria, even during long-term applications. This allows treatment of atopic dermatitis flares with reduced topical corticosteroid application [349,350,351]. Importantly, to date, no bacterial resistance to this endolysin has been identified [348,349,350,351], even with long-term daily usage to treat chronic and recurrent *S. aureus* infections [212].

#### 5.4.1. Staphefekt SA.100 In Vitro Analysis

An in vitro analysis with the use of a lysis assay showed that Staphefekt (30 mg/mL) was active against 28 MSSA and 8 MRSA strains (58.6% vs. 54.1% OD reduction, respectively in comparison to untreated controls) [349]. *S. epidermidis*, *S. hominis*, *Staphylococcus haemolyticus*, and *Staphylococcus lugdunensis* strains, on the other hand, were only minimally impacted (1%–15% reduction) [349]. Further analysis also presented equivalent susceptibility for MRSA and MSSA strains (2 to 3 log CFUs/mL reduction in culture within 4 h and no discrepancies in MIC analysis).

#### 5.4.2. Staphefekt SA.100 Clinical Trial

In 2016, Staphefekt SA.100 was subject to a phase I/II clinical trial (Table 2; ClinicalTrials.gov identifier NCT02840955) focusing on its impact on *S. aureus* infections, the skin microbiome, disease severity, quality of life, and corticosteroid co-treatment (triamcinolone). One hundred participants with moderate-to-severe atopic dermatitis were treated with a cetomacrogol-based cream for 12 weeks [189], with the drug well tolerated by patients. There currently is a lack of information about the impact of the drug on corticosteroid usage and dosing, however, mainly due to patient lack of compliance with the combination treatment, hampering the study size. Moreover, 21 Staphefekt-naive healthy human donors were tested and pre-existing IgG antibodies recognizing Staphefekt epitopes were identified. The authors suggest that this is probably due to daily *S. aureus* exposure, including, presumably, also to their phages and lysins. Staphefekt is now in preparation for a phase III clinical trial to evaluate its efficiency against *S. aureus* infections found in association with eczema in comparison to topical antibiotics.

#### 5.4.3. Staphefekt SA.100 Case Study Series

Totté et al. [212] presented three case studies based on Staphefekt SA.100 topical treatment of dermatosis associated with *S. aureus* colonization. In the first case, a 23-year-old male was reluctant to use oral antibiotics and instead was treated with Staphefekt SA.100 twice daily for two weeks, with a reported (p. 20) “strong decrease of inflammatory symptoms that started within a few days.” The symptoms returned within 1 week of cessation of treatment, however, suggesting that only control had been achieved rather than elimination of the *S. aureus*. In the second case, a 63-year-old male was treated for impetigo with various antibiotics, with flucloxacillin resulting in clinical improvement, although this improvement persisted only over the course of treatment. The symptoms were abolished over a subsequent 12-week treatment with Staphefekt, as combined with the anti-inflammatory betamethasone, although again *S. aureus* was not eliminated. The third case also involved a 23-year-old male who had been treated as well with flucloxacillin, resulting in the temporary improvement of symptoms. As with the first two cases, substantial improvement was seen with subsequent Staphefekt treatment. The authors concluded that (p. 22), “We believe that Staphefekt induces a clinically relevant reduction of *S. aureus* rather than a total eradication.” Micreos, which provides this endolysin commercially, also reports a customer satisfaction rating of over 80% in their customer questionnaires [189].

### 5.5. Development of Enzybiotics Targeting Gram-Negative Bacteria towards Clinical Trials

Currently, to our knowledge, there are no registered clinical trials with the use of phage-based enzybiotics targeting Gram-negative pathogens. Nevertheless, several research groups have applied synthetic biology approaches to engineer phage PGHs to tackle Gram-negative pathogens. The difficulties in phage lysin-based treatments against Gram-negative pathogens occur mainly due to the problem of bacterial outer membrane penetration. Hybrid proteins are, therefore, created by the fusion of PGHs with different outer membrane permeabilizing peptides. Alternatively, completely new chimeric proteins can be created with new features with the use of, e.g., the VersaTile shuffling method [309,310].

In Europe, Lysando AG has prepared Artilysins^®^-based drugs with antibacterial properties against Gram-positive and Gram-negative pathogens. Since 2014, they developed Artilysins^®^ against *Campylobacter* spp., *S. aureus*, *Staphylococcus* spp., *P. aeruginosa*, *Streptococcus uberis*, *Salmonella* spp., *E. coli*, *Vibro* spp., *Neisseria gonorrhoeae*, *A. baumannii*, *K. pneumoniae*, *Cutibacterium acnes,* as well as several with dual activity, targeting *P. aeruginosa* and *Enterobacteriaceae*, *Enterococcus faecalis* and *E. faecium*, *Streptococcus dysgalactiae* and *S. agalactiae*, or *Enterococcus* spp. and *Streptococcus* spp. [352]. Lysando AG is currently working toward obtaining licensees in animal feed areas as well as starting patient treatment with Artilysins^®^-based wound care sprays (Medolysin^®^) with antibacterial and wound healing properties.

According to the Lysando AG website, the recruited patients were treated daily for more than 30 days or every 2–3 days up to a maximum of five applications. The wound healing process was improved immediately post-application in 90% of the subjects, leading to up to 40% completely healed wounds within 30 days. Moreover, the patient life quality was improved, including pain and inflammation reduction. Additionally, the chronic MRSA decubitus infection of one coma patient was completely healed after 27 months of treatment, with the first signs of wound healing beginning within 2 weeks of the start of treatment [352]. However, clinical publications to support these claims do not exist.

## 6. Advantages and Challenges

Phage-based enzybiotics present a promising alternative antimicrobial therapy. Currently, the most progress has been achieved with PGHs, which provide both activity and high efficiency against many clinically relevant pathogens. They have distinctive modes of action, relative to standard antibiotics, and can be applied as stand-alone therapies as well as in combination with other antimicrobials. Importantly, the possibility of the occurrence of resistance appears to be low, although some cases of acquired resistance have been observed with engineered endolysins, as documented in Section 5.1.3. Furthermore, PGHs often are highly specific to their bacterial targets, having therefore a minimal impact on the microbiome in contrast to many antibiotics. Therapy with phage-derived enzybiotics can also be used to tackle not only planktonic but also biofilm-embedded bacteria.

In comparison to whole phages, protein-based enzybiotics do not require replication in association with their hosts, which often are pathogenic bacteria. This is especially problematic with large-scale industrial phage production, where additional containment measures thereby need to be assured. In contrast, methods of recombinant protein production are well developed, and some of these approaches do not require even bacterial involvement. Phage replication in the presence of bacterial hosts can also be problematic in terms of the repeatability of the phage titers to which targeted bacteria are exposed during treatments. Additionally, there is a risk that phages may mutate and, at least in theory, change specificity as well as promote gene exchange between bacteria. All of these issues can be avoided with the use of protein-based enzybiotics.

Both phages and enzybiotics can induce immune responses and, often, the production of anti-phage or anti-enzyme antibodies is observed. Nevertheless, it seems that host immune system responses do not have substantial negative impacts on the therapeutic outcomes of either phage or enzybiotic treatments. Moreover, the duration of the treatments, dosages, delivery routes, and immune status of patients may influence the immune response, which often is case-specific [53].

PGHs can tackle Gram-positive and Gram-negative bacteria, although, as discussed in Section 5.5, targeting Gram-negative pathogens is not always straightforward as it requires overcoming the outer membrane barrier. Currently, the most explored tactic is protein engineering, allowing for PGH fusion with outer membrane permeabilizing peptides. This approach also opens the possibility for various modifications of protein properties and the shuffling of PGH’s domains, increasing the diversity and specificity of the available antibacterial proteins.

The phage enzybiotic field is new and there is a limited amount of data concerning their safety, pharmacokinetics, and pharmacodynamics, e.g., [230,255,271]. Over the course of treatment, there is a risk, e.g., of releasing pathogen-associated molecular patterns (PAMPs), such as LPS, from bacteria upon their lysis [53,353]. This can lead to severe side effects, including endotoxic shock. The results from animal models and first clinical trials, however, are both promising and present safe profiles for PGHs. The N-Rephasin^®^ clinical trials data are concerning at this moment, however, although without publication and discussion from the company side it is difficult to assess the outcomes of these trials, including whether endolysin itself leads to severe side effects or if other factors played a role.

There are also other bottlenecks in enzybiotic development. These include delivery routes and methods. In the literature, most phage-derived enzymes are applied only topically. Systemic, oral delivery, or topical delivery into the lungs are more challenging. Enzymatic conformations and activities can be affected by changes in pH in the digestive system, by the activity of different enzymes, such as proteases or peptidases, or the activity of alveolar macrophages [53,354,355]. Patients alternatively can be exposed to secondary bacterial infections when a drug is administrated intravenously. To overcome these obstacles, several different approaches are currently under investigation, such as liposome encapsulation [356], encapsulation within Poly (*N*-isopropylacrylamide) nanoparticles [357], or PGH fusion with bacteriocins that allow these enzybiotics to cross the outer membrane (“lysocins” [358]). Important consideration is also necessary regarding the protein size as this can limit drug delivery to bacterial infections, e.g., as involving penetration through nasal mucosa or gut epithelia, or deposition into lungs [359]. This is especially a concern when proteins are engineered and fused with additional peptides or domains as this has the effect of increasing overall protein size.

Finally, an important hurdle is also approval from drug regulatory agencies, such as the EMA (European Medical Agency) in Europe or the FDA (Food and Drug Administration) in the USA. PGHs and PSDs are produced via recombinant DNA technologies and are also often engineered, making routes toward approval as well as launching clinical trials more complicated. To date, there is only one endolysin-based product, Staphefekt SA.100, which is available in Europe as a medical product. Its regulatory approval, however, is suggestive of a bright future for the clinical use of enzybiotics to treat common bacterial infections.

## 7. Conclusions

In the context of the worldwide antibiotic crisis, new alternative therapies are in grave need. Phage-derived enzybiotics represent a promising and novel class of therapeutics for human and animal applications. They demonstrate strong anti-biofilm and antibacterial properties in vitro, in vivo, and in human studies. They are, in general, considered to be safe, non-toxic agents that are active against multi-drug-resistant Gram-negative and Gram-positive pathogens as well as persister cells, with low probability of bacterial resistance occurrence, the latter especially for peptidoglycan hydrolases (PGHs). Enzybiotics can also be applied as adjuvants or co-treatments to standard-of-care antibiotics. Nevertheless, additional research is still necessary toward further improvement of these therapies as well as their regulatory approval for clinical use.

## Figures and Tables

**Table 1:** Phage-derived polysaccharide depolymerases efficacy in vivo.

Enzyme	Pathogen	Animal	Infection	Inoculum	Dosing	Results	Ref.
P22 tailspike protein (P22sTsp) recognizing LPS	*Salmonella enterica* serovar Typhimurium	Leghorn chicks	Intestinal colonization	Oral gavaging, 300 µL PBS containing 10^4^ to 10^7^ CFU	Oral gavaging, 300 µL in 10% BSA containing 30 mg; 3 doses: 1st 1 h post-infection, 2nd and 3rd dose given in 24 h intervals	100-fold reduction of *Salmonella* colonization in the gut as well as reduced liver and spleen penetration; *Salmonella* motility was impaired	[150]
Dep-ORF8 targeting capsular serogroup A	*Pasteurella multocida* capsular serogroup A	BALB/c mouse model	Systemic infection	IP injection of 100 µL containing 80 CFU	3 treatment groups: IP injection of 100 µL containing 36 µg at 6 h (group 1), 12 h post-infection (group 2), and 12 h post-infection, and then once daily for 5 days (group 3)	Treatment: group 1 showed ~70%, and 50% survival within 3 and 5 days, respectively; group 2 showed 70%, 50%, and ~35% survival within 3, 5, and 12 days, respectively; group 3 showed ~80%, 70% survival within 4 and 6 days, respectively; control group 100% mortality within 5 days	[151]
gp49, O-specific polysaccharide lyase	*Pseudomonas aeruginosa*	*Galleria mellonella* (Wax moth larvae)	Hemocoel infection	Injection into the last pro-leg of 10 CFU	Pretreatment: 1h incubation of bacteria with 50 µg/mL; treatment: 5 or 50 µg/mL was injected 15 min post-infection	Pretreatment: 24 h post injection, 50% of larvae survived (~30% more than in the control); 35% larvae also survived to the end of the experiment (>72 h); treatment: 24 h post-treatment, the larvae survival rate was at least 20% higher compared to the control, independent of gp49 concentration; 20% of larvae survived up to 72 h with treatment, while 100% of control group died 48 h post injection	[105]
depoKP36 targeting KP36 capsule	*Klebsiella pneumoniae*	*G. mellonella* (Wax moth larvae)	Hemocoel infection	Injection into the last pro-leg of 10 µL containing 10^7^ CFU	Pretreatment: bacteria were pretreated with depoKP36 (280 µg/mL) for 2 h before infection; treatment: depoKP36 was administered 5 min post-infection	Pretreatment: 77% of larvae were saved within 24 h, and 47% and 43% after 48h and 72 h, respectively; treatment: survival increased up to 40%, 30%, and 20% after 24 h, 48 h, and 72 h post-treatment, respectively; 100% of untreated larvae died	[132]
Dp42 targeting capsular polysaccharide type KN1	*K. pneumoniae*	BALB/c mouse model	Systemic infection	IP injection of 2 × 10^7^ CFU	Prevention: IP injection of 200 µL containing 0.25 mg/mL 6 h before bacterial infection; pretreatment: 0.25 mg/mL for 30 min; treatment: IP injection of 200 mL containing ~50 mg 30 min post-infection	Prevention: 100% survival within 96 h post-infection, while 100% of control group died within 9 h; pretreatment: 1 mouse died (12.5%) 54 h post-infection, while 100% of control group died within 12 h; treatment: identical to pretreatment results.	[152]
K64dep targeting K64 capsular type polysaccharides	*K. pneumoniae*	BALB/cByl mouse model CP treated, 200 mg/kg IP injections in 2 days intervals	Systemic infection	IP injection of 6 × 10^6^ CFU	IP injection with 150 μg, 37.5 μg, or 18.75 μg at 1 h, 8 h, and 24 h post-infection	100% survival with 18.75 µg dose applied 1 h post-infection; in control group, 100% mortality was observed; 150 µg dose applied 8 h post-infection had no effect; no K64dep-related toxicity was observed as well as no changes in liver, kidney, and spleen histopathology; treatment sensitizes carbapenem-resistant K64 to serum killing in vitro as well as increased its susceptibility to neutrophil killing (~40% improved killing)	[153]
Endosialidase E (endoE)	*Escherichia coli* producing K1 antigen	Neonatal rats	Intestinal colonization and *E. coli*-related bacteremia	Oral administration of 20 μL containing 2 to 6 × 10^6^ CFU	IP injection of 20 µg on days 1, 2, 3, 4, and 5 post-infection	No direct effect on *E. coli* viability but pathogen is sensitized to complement system killing; single dose on day 1 of endoE prevents the death of infected pups and *E. coli* invasion of the bloodstream; 80–100% survival in comparison to 0–10% survival in untreated control	[133]
Endosialidase E (endoE)	*E. coli* producing K1 antigen	Neonatal rats	Intestinal colonization and *E. coli*-related bacteremia	Oral administration of 20 μL containing 2 to 6 × 10^6^ CFU	IP injection of 0.125–20 µg range on days 1 post-infection	Minimal dose of 0.25 µg prevented death of at least 80% of rats; treatment sensitizes *E. coli* to serum killing in vitro, and improved macrophage ingestion of *E. coli*	[134]
Dep6, O91-specific polysaccharide depolymerase	Shiga toxin-producing *E. coli*	BALB/c mouse thigh model	Systemic infection	Injection near the right thigh of 100 μL containing 2.4 × 10^8^ CFU	Dose: 100 μL containing 0.3 μg/μL; toxicity: IP injection; prophylactic: delivery 3 h prior to infection; simultaneous treatment: delivery at the same time as bacterial inoculum; delayed treatment: delivery 3 h post-infection	Toxicity analysis: no pathological changes in liver, kidney, or small intestine observed; pretreatment: 100% survival; simultaneous treatment: 83% survival; delayed treatment: 33% survival; significant reduction in the levels of proinflammatory cytokines was observed at 24 h post-infection	[138]
Capsule depolymerases active against three different capsule types: K1, K5, and K30	*E. coli*	NIH Swiss Mouse thigh model	Systemic infection	Injection into thigh of 100 µL containing 1 to 4 × 10^8^ CFU	Injection of 100 µL PBS containing 0, 2, 5, or 20 µg doses, 30 min post-infection; different depolymerases tested	Toxicity: no toxicity observed; treatment: control group did not survive, whereas most mice were rescued by treatment with 20 µg dose per mouse; effective doses of K1F and K1H enzymes were between 2 µg (both partially rescuing) and 5 µg (both rescuing 100% mice) per mouse; for K5, the effective dose was between 2 and 20 µg per mouse; K30 gp41 rescued mice at the higher dose tested (20 µg per mouse); a mixture of K30 gp41 and K30 gp42 yielded the same survival outcome as K30 gp41 alone	[122]
ϕAB6 targeting capsular polysaccharide	*Acinetobacter baumannii*	Zebrafish	Systemic infection	Injection through cloaca of 1 to 4 × 10^7^ CFU	Injection through cloaca of 20 μL protein (1 μg/μL), 30 min post-infection	Treatment: survival rate was significantly improved (80%) compared with untreated control (10%); toxicity: none observed	[154]
Dpo48 capsule depolymerase	*A. baumannii*	*G. mellonella* (Wax moth larvae)	Hemocoel infection	Injected into the last pro-leg of 10 µL PBS containing 10^6^ CFU	Pretreatment: 50 µg/mL for 1 h; treatment: Injection of 10 µL PBS containing 5 µg 5 min post-infection	Pretreatment: 100% survival, while, in control group, ~65% and 84% of larvae died within 24 h and 72 h, respectively; treatment: 76% survival, while, in control group, ~65% and 84% of larvae died within 24 h and 72 h, respectively	[155]
Dpo48 capsule depolymerase	*A. baumannii*	BALB/c mice model	Systemic infection	IP injection of 10^7^ CFU	IP injection of 200 µL PBS containing 50 µg 2 h post-infection	100% mice treated survived and appeared healthy for 7 days, while 100% of the untreated control died within 24 h due to peritoneal sepsis; bacterial count in tissue and organs was significantly reduced with treatment 6 h post-infection in comparison to control group	
		BALB/c mice model, IP injection of CP (300 mg/kg) in 200 µL PBS, 3 days before infection	Systemic infection	IP injection of 10^7^ CFU	IP injection of 200 µL of PBS containing 50 µg 2 h post- infection	100% of mice treated survived and appeared healthy for 7 days, while 100% of untreated control died within 24 h due to peritoneal sepsis	
K2 capsular depolymerase	*A. baumannii* capsular type K2	*G. mellonella* (Wax moth larvae)	Hemocoel infection	Injection into the last pro-leg of 5.5 µL of 20 mM HEPES containing 10^6^ CFU	Pretreatment: bacteria pretreated with protein for 2 h; treatment: injection of enzyme 30 min post-infection; in both scenarios, a range of protein dosages were used (0.25 g, 0.5 g, and 3 g/larvae)	No toxicity, 100% survival of larvae; pretreatment: untreated control group survival rate was 25%, 20%, and 10% after 24, 48, and 72 h, respectively; in group with pretreatment after 72 h, 53%, 69%, and 88% of larvae survived using 0.25 g, 0.5 g, and 3 g pretreatments; treatment: only 35%, 22%, and <15% larvae survived in untreated control after 24 h, 48 h, 72 h, respectively, while 73%, 40–76%, 56–70% survived with treatment; K2 depolymerase is highly refractory to resistance development	[156]
		BALB/c mouse model, IP injection of CP (100 mg/kg), 4 and 1 day before infection	Systemic infection	IP injection of 10^7^ CFU	IP injection with 50 µg dose 1 h post-infection	20 h post-infection control group had to be euthanized, while in a treatment group 90% mice had survived, decreasing to 60% at 42 h post-infection	

Abbreviations: (CP) cyclophosphamide, (IP) intraperitoneal.

**Table 2:** Clinical trials of phage lytic enzymes.

Descriptor	Company	Type	Route	Phase	#	Start	Status	Registry #	Protocol and Observations	Ref.
P128 (StaphTAME)	GangaGen	Ectolysin	IN	I/II	74	2012	Completed	NCT01746654	Type: randomized, double-blind, placebo-controlled study; goal: (1) evaluation of safety, tolerability via single or multiple doses (3 doses/day for 5 days) of 0.1 mg, 0.3 mg, and 1mg concentrations of P128, administrated intranasally to healthy individuals; (2) evaluation of safety, tolerability, and efficacy of P128 in chronic kidney disease patients or any patients who are nasal carriers of *S. aureus* or MRSA strain with single dose or 3 escalating concentrations of P128; Initial results: drug was well tolerated; results: reduction of nasal carriage	NA
N-Rephasin^®^ SAL200 (SAL-1, tonabacase)	iNtRON Biotechnology	Endolysin	IV	I	36	2013	Completed	NCT01855048	Type: randomized, double-blind, placebo-controlled study; goal: evaluation of safety, pharmacokinetics, and pharmacodynamics of single intravenous dose of SAL-1 at various concentrations: 0.1 mg/kg, 0.3 mg/kg, 1 mg/kg, 3 mg/kg, 10 mg/kg, administrated to healthy male individuals; results: no severe side effects observed	[157]
				II	25	2017	Terminated ^1^	NCT03089697	Type: randomized, double-blind, placebo-controlled study; goal: evaluation of safety and efficacy of SAL-1 (3mg/kg), administrated once a day intravenously to individuals with persistent *S. aureus* bacteremia; results: serious adverse effects occurred (2/12 patients, 16.67% of test group), including pneumonia (one patient, 8.33% of test group) and respiratory failure (one patient, 8.33% of test group), as well as several other minor adverse events (10/12 patients, 83.33% of the test group), e.g., anemia, chills, back pain, headache, gastrointestinal disorders	NA
Lysin CF-301 (PlySs2, exebacase)	ContraFect	Endolysin	IV	I	20	2015	Completed	NCT02439359	Type: placebo-controlled, dose-escalating study; goal: evaluation of safety and tolerability of single intravenous dose of CF-301; healthy male and female individuals; results: CF-301 has a safe profile with no side effects observed	[266,267,268,269,270]
				II	121	2017	Completed	NCT03163446	Type: multicenter, randomized, double-blind, placebo-controlled study; goal: evaluation of safety, tolerability, efficacy, and pharmacokinetics of CF-301; study performed in addition to standard-of-care antibacterial therapy; adult individuals with bloodstream infections (bacteremia), including endocarditis	[325]
				III	348	2019	Ongoing	NCT04160468	Type: randomized, double-blind, placebo-controlled study; goal: evaluation of the efficacy and safety of a single dose of Exebacase in addition to standard-of-care antibacterial therapy; adult individuals with bloodstream infections (bacteremia), including endocarditis	NA
Staphefekt SA.100	Micreos	Endolysin	T	I/II ^1^	100	2016	Completed	NCT02840955	Goal: evaluation on disease severity and skin microbiome; individuals with atopic dermatitis; results: no side effects observed, decrease in bacterial burden	[189]

Abbreviations: (#) number of participants, (IN) intranasally, (IP) interperitoneally, (IV) intravenously, (T) topically, (NA) not available. ^1^ Enrollment into this study was terminated by the sponsor prior to completion of the study.
